# Gastric Volvulus After Nissen Fundoplication: A Case Report

**DOI:** 10.7759/cureus.97671

**Published:** 2025-11-24

**Authors:** Maria Vasconcelos, Clara Rocha, Frederico Afonso, Sofia Morais

**Affiliations:** 1 General Surgery, Hospital Prof. Doutor Fernando Fonseca, Amadora, PRT

**Keywords:** gastric volvulus, hiatal hernia, mesenteroaxial gastric volvulus, nissen fundoplication, upper gastrointestinal surgery

## Abstract

Gastric volvulus is a rare condition in which there is an abnormal rotation of the stomach, which can be life-threatening. Most cases occur in patients with a hiatal hernia or other diaphragmatic defects. Gastric volvulus after Nissen fundoplication has been rarely reported, with several proposed mechanisms.

We present the case of a 53-year-old woman with a history of previous Nissen fundoplication who presented with nausea and vomiting due to a mesenteroaxial gastric volvulus caused by an adhesion. Adhesiolysis and volvulus reduction were performed, along with gastropexy. The postoperative course was complicated by septic shock due to bacterial translocation, requiring intensive care unit (ICU) admission. The patient subsequently recovered fully.

Gastric volvulus may present acutely or with more intermittent symptoms. Diagnosis is often challenging, as Borchardt's triad is not always present. Endoscopy may be a valuable tool in diagnosis and initial management, but definitive treatment is surgical. In this case, the fundoplication remained intact with the volvulus being attributed to adhesions and gastric laxity. Despite prompt intervention and the absence of ischemia, the patient still developed septic shock and required ICU admission, underscoring the importance of early recognition and management.

Gastric volvulus can occur after Nissen fundoplication even without hernia recurrence. Prompt diagnosis and intervention are crucial to avoid complications. In stable patients with a viable stomach, surgery should aim to restore anatomy and prevent recurrence.

## Introduction

Gastric volvulus is a rare condition in which there is an abnormal rotation of the stomach. The rotation can be along the stomach's longitudinal (organo-axial) or transverse (mesenteroaxial) axis [1]. While most cases of volvulus are organo-axial and associated with diaphragmatic anomalies, mesenteroaxial volvulus represents around 30% of cases [2]. In mesenteroaxial volvulus, the pylorus is rotated anteriorly and superiorly to the gastroesophageal junction [3].

Gastric volvulus may also be classified based on etiology. Primary gastric volvulus is generally due to abnormalities of the gastric ligaments with failure of gastric fixation. Secondary gastric volvulus is more common and is defined as volvulus resulting from other anatomic or functional abnormalities such as paraesophageal hernias or diaphragmatic eventrations [2].

Gastric volvulus after Nissen fundoplication has been rarely reported. A Nissen fundoplication is a commonly performed antireflux procedure, in which the fundus of the stomach is wrapped around the lower esophagus. When volvulus occurs in this setting, proposed mechanisms include ligamentous laxity, intrathoracic migration of the stomach, or the development of postoperative adhesions [4].

Despite the rarity of this entity, early diagnosis is critical to avoid life-threatening complications such as strangulation and perforation.

In this article, we present the case of a patient with a history of laparoscopic Nissen fundoplication performed one year prior, who presented with signs of upper gastrointestinal obstruction due to mesenteroaxial gastric volvulus.

## Case presentation

**Figure 1:**
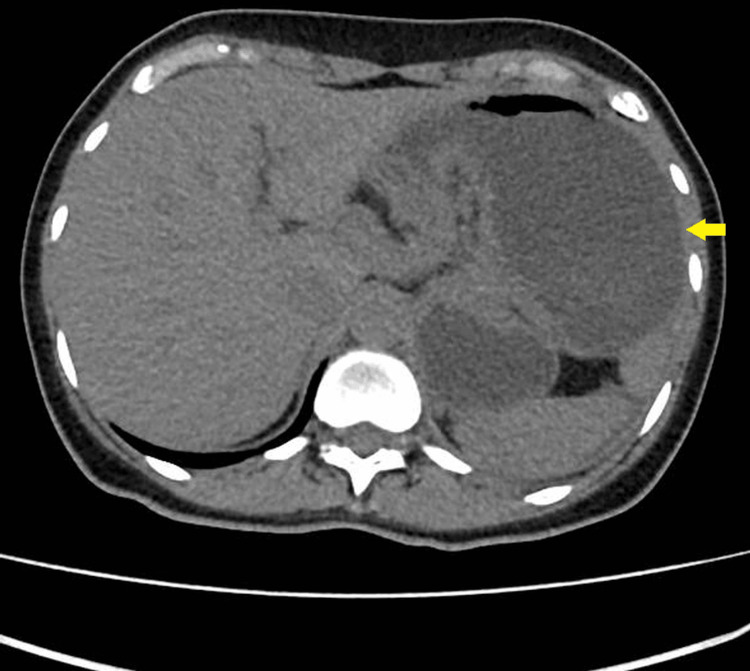
Axial view of the CT scan of the abdomen and pelvis demonstrating gastric volvulus CT: computed tomography

**Figure 2:**
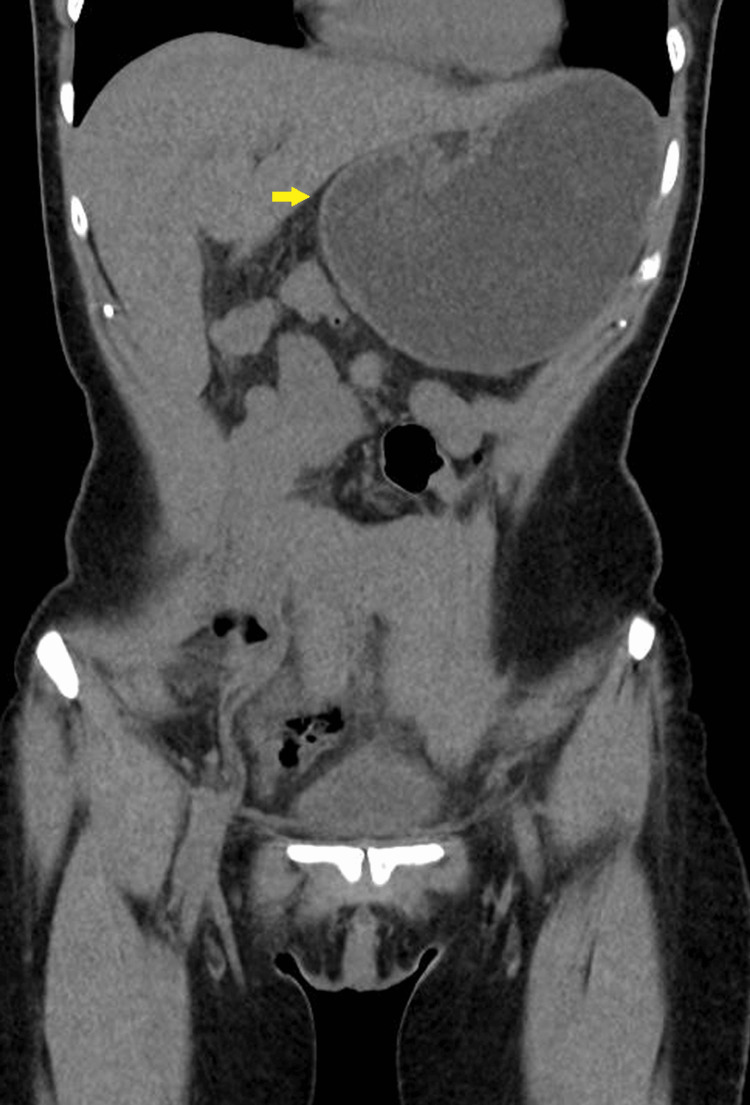
Coronal view of the CT scan of the abdomen and pelvis demonstrating gastric volvulus: the arrow shows the abnormal superior-anterior position of the pylorus CT: computed tomography

A 53-year-old female patient with a history of hiatal hernia repair with laparoscopic Nissen fundoplication presented to the emergency department with abdominal pain, nausea, and vomiting. In the year prior, she reported symptoms of bloating and unspecified weight loss, with occasional vomiting episodes. Examination revealed a distended abdomen with tenderness in the upper quadrants. Vital signs were notable only for mild sinus tachycardia with a heart rate of 110 bpm. Initial laboratory results showed no relevant changes (Table 1). Admission arterial blood gas (ABG) showed no hyperlactacidemia. Computed tomography (CT) of the abdomen and pelvis (Figure 1 and Figure 2) showed significant gastric distention caused by mesenteroaxial volvulus of the stomach.

**Table 1:** Admission laboratory values

Parameters	Patient results	Reference ranges
Hemoglobin	14.4 g/dL	12-15 g/dL
White blood cell count	10.2 × 10^9^/L	4.10 × 10^9^/L
Neutrophils	6.7%	2-7%
Lactate	0.9 mmol/L	<1.8 mmol/L
Sodium	144 mmol/L	136-145 mmol/L
Potassium	3.15 mmol/L	3.5-5.10 mmol/L
Creatinine	0.72 mg/dL	0.5-0.9 mg/dL
C-reactive protein	0.62 mg/dL	<0.5 mg/dL

A 14 Fr nasogastric tube was placed with drainage of approximately 1.5 L of gastric contents. An esophagogastroduodenoscopy (EGD) was performed, which confirmed the gastric volvulus, with torsion of the gastric body and antrum. There was no evidence of mucosal ischemia, but detorsion was not possible endoscopically.

The patient underwent surgery the following day. Findings at diagnostic laparoscopy revealed signs of previous Nissen fundoplication with near-complete mobilization of the upper greater curvature. There was a mesenteroaxial volvulus of the stomach due to an adhesion between the antrum and the right liver lobe. At this point, due to difficulty in achieving adequate detorsion of the stomach, the procedure was converted to an open laparotomy. The adhesion was divided, and the volvulus reduced, restoring normal anatomy. Gastropexy was performed with non-absorbable stitches between the stomach and the left hemidiaphragm and the anterior abdominal wall. The nasogastric tube was left in place for continued gastric decompression.

**Figure 3:**
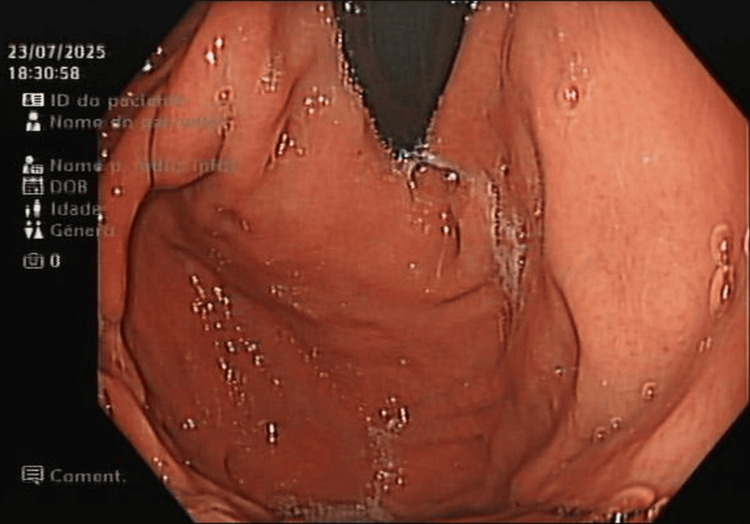
Repeat EGD Repeat EGD at six months demonstrating normal post-fundoplication anatomy. Unfortunately, the initial EGD images were lost due to a power outage. EGD: esophagogastroduodenoscopy

The postoperative course was complicated by hypotension and oliguria, assumed to be in the context of septic shock due to bacterial translocation. Blood cultures were positive for *Escherichia coli* and *Citrobacter freundii*. The patient was admitted to the intensive care unit (ICU) and quickly improved under vasopressor support and directed antibiotherapy with piperacillin-tazobactam. The remaining postoperative course was uneventful, and the patient was discharged home on postoperative day 9. At her follow-up visits, at one and six months, she reported no symptoms. A repeat EGD at six months demonstrated normal post-fundoplication anatomy (Figure 3).

## Discussion

Gastric volvulus can present acutely with complete obstruction or more chronically with intermittent symptoms. It can be a life-threatening condition, requiring timely detection to avoid life-threatening complications such as gastric ischemia and perforation.

Some patients with gastric volvulus present with Borchardt's triad: sudden severe epigastric pain, intractable retching, and inability to pass a nasogastric tube [5]. However, this triad is often not present, as seen in our patient, who was able to vomit, in which it was possible to place a nasogastric tube. Therefore, diagnosis can be challenging and is often delayed.

Acute gastric volvulus is a surgical emergency even in patients who are stable, due to the risk of progression to gastric ischemia and perforation. EGD can be valuable in the early management of gastric volvulus as it can permit not only gastric decompression and placement of a nasogastric tube but also visualization of the gastric mucosa. Also, in some cases, EGD may reduce the volvulus and relieve the obstruction, allowing semi-elective surgical repair [2]. In our case, EGD was not effective in reducing the volvulus, and as such, surgery was anticipated.

Our patient had a mesenteroaxial volvulus, which we hypothesize was conditioned by laxity of the stomach and an adhesion of the antrum to the liver; both are results of her previous surgery. Her previous intermittent symptoms suggest a chronic progression of the volvulus, probably due to prior mobilization of the greater curvature. This laxity might have aided the formation of the mentioned adhesion and evolution to a scenario of complete obstruction. Unlike other case reports in the literature [4,6-8], the fundoplication was found to be intact at the time of surgery, without hernia recurrence. Reviewing the literature, we found three other cases of volvulus post-Nissen fundoplication without hernia recurrence; one of them was also due to adhesions [9-11].

In most cases reported in the literature, the initial exploration and subsequent surgical repair were performed laparoscopically [4,7,9], with conversion to laparotomy when necessary [6]. The specific surgical approach depended on the underlying cause of the volvulus, with management often involving adhesiolysis or revision of a previous fundoplication. In one reported case, total gastrectomy was required due to irreversible gastric ischemia [4]. Following reduction of the volvulus, most authors described performing some form of gastric fixation, either by placing a gastrostomy tube [9] or by performing a gastropexy [6,7].

Although gastric ischemia was absent, and despite proper antibiotic prophylaxis, the patient still suffered multiorgan dysfunction, requiring ICU admission. This highlights the danger of this condition and the need for prompt diagnosis and management.

## Conclusions

Gastric volvulus can occur in patients with previous Nissen fundoplication, even in the absence of hiatal hernia recurrence. Timely diagnosis is difficult, but it avoids life-threatening complications such as gastric strangulation and perforation. In stable patients and in the absence of gastric necrosis, the goals of surgical treatment are restoring the normal anatomy and preventing future stomach rotation; in this case, this was achieved with volvulus reduction and gastropexy.
